# Management of retroperitoneal appendiceal perforation: a case report

**DOI:** 10.1093/jscr/rjae069

**Published:** 2024-02-13

**Authors:** Jinliang Li, Feng Li, Jun Li, Chunlei Wang, Yubao Sun, Xiaowei Bian

**Affiliations:** Department of Emergency Surgery, Weifang People’s Hospital, Weifang, Shandong 261000, China; Department of Emergency Surgery, Weifang People’s Hospital, Weifang, Shandong 261000, China; Department of Emergency Surgery, Weifang People’s Hospital, Weifang, Shandong 261000, China; Department of Emergency Surgery, Weifang People’s Hospital, Weifang, Shandong 261000, China; Department of Emergency Surgery, Weifang People’s Hospital, Weifang, Shandong 261000, China; Department of Emergency Surgery, Weifang People’s Hospital, Weifang, Shandong 261000, China

**Keywords:** appendix, retroperitoneum, perforation

## Abstract

Retroperitoneal appendiceal perforation presents unique challenges in surgical management due to the complex nature of the retroperitoneal space. We present a case of a 57-year-old male with retroperitoneal appendiceal perforation, characterized by the presence of a large amount of gas in the retroperitoneal space. Emergent laparoscopic surgery was performed to address the retroperitoneal involvement. In retroperitoneal appendiceal perforation, surgical intervention and postoperative drainage are of great significance to prevent septic shock. The interconnectedness of the retroperitoneal space with other body regions is highlighted, underscoring the potential for severe complications. This case emphasizes the need for a tailored approach to managing retroperitoneal appendiceal perforation, preventing potential complications associated with this condition.

## Introduction

Appendicitis is a common surgical illness that often causes acute abdominal pain and peritonitis. In cases where the appendix perforates, extraluminal air or abscess can be detected in the peritoneum. However, the consequences of appendiceal perforation vary depending on the position of the appendix. Retroperitoneal perforation of the appendix is particularly significant. Unlike intraperitoneal perforation, pus cannot be easily collected and suctioned in retroperitoneal perforation. What’s more, the retroperitoneum contains a large amount of fat tissue, making it difficult to perform abscess drainage. In this case report, we present a situation where approximately half of the retroperitoneum was affected by appendiceal perforation.

## Case presentation

**Figure 1:**
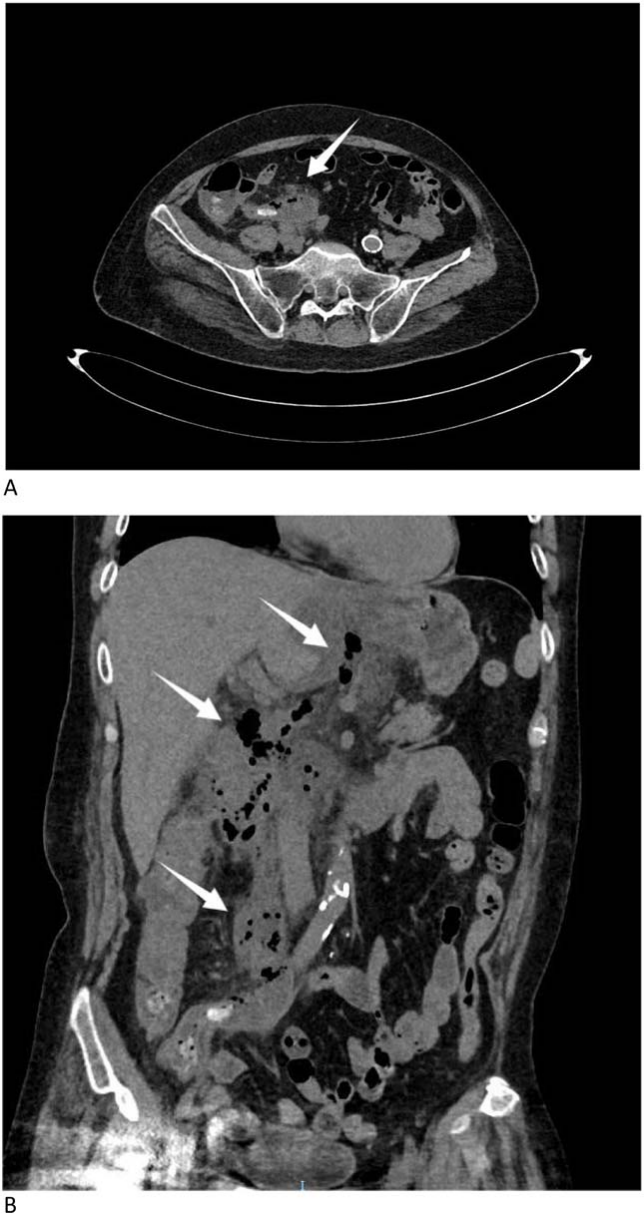
(A) CT scan identified an appendicolith with diffuse infiltration (arrow). (B) CT scan showed a large amount of gas in the retroperitoneal space, extending from the cecum along the mesentery to the stomach (arrows).

A 57-year-old male presented to the emergency department with gradually increasing right-sided abdominal and back pain, accompanied by a fever of 39.6°C for the past 8 days. He was initially evaluated at a local hospital where ultrasound revealed perforation. He was subsequently admitted to our hospital. Physical examination revealed abdominal tenderness. The patient had no significant medical history. Laboratory tests showed a white blood cell count of 20.8 × 10^9^/L and elevated C-reactive protein levels of 147.1 mg/L, indicating sepsis. Other laboratory data also showed hyponatremia, hypokalemia, and hypoproteinemia due to poor appetite following the onset of symptoms. Immediate computed tomography (CT) scan identified an appendicolith with diffuse infiltration (Fig. 1A), and a large amount of gas was observed in the retroperitoneal space, extending from the cecum along the mesentery to the stomach (Fig. 1B). The patient was diagnosed with retroperitoneal perforation.

**Figure 2:**
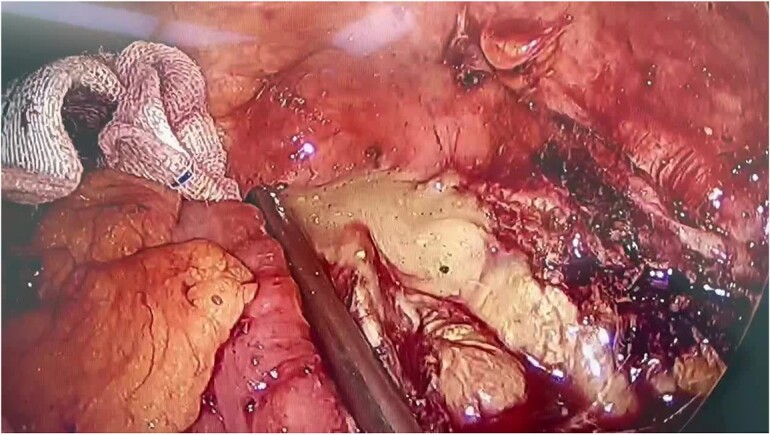
During the operation, a significant accumulation of pus was revealed.

The patient underwent emergent laparoscopic surgery. During the operation, the peritoneum was found to be clean, except for an abscess located behind the lesser omentum. The appendix was buried behind the terminal part of the ileum, making it difficult to recognize due to the loss of its normal shape. The appendiceal wall was scattered in the pus. During the surgical procedure, we gained access to the retroperitoneal abscess by opening the right paracolic gutter, which revealed a significant accumulation of dark yellow pus (Fig. 2). Appendicectomy and necrosectomy were performed in the retroperitoneal space. Necrotic tissue behind the cecum, small intestine, and stomach was removed. Four drainage tubes were placed at the end of the surgery, two in the peritoneum and two in the retroperitoneum. Necrotic tissue was observed in the drainage tubes, particularly in the retroperitoneal ones, during the first 7 days. The amount of necrotic tissue decreased over time, and the peritoneal drains were removed first. After 13 days post-surgery, all drainage tubes were removed. On the 15th day of admission, the patient’s laboratory data improved, and he was discharged from the hospital.

## Discussion

Acute appendicitis is the most common abdominal emergency, with an annual incidence of 11/10000 population [1]. Appendix perforation is a significant complication that increases the morbidity and mortality rate in operated patients to ~5% [2, 3]. The appendix is typically located in the intraperitoneum, either anterior or retrocecal. Approximately 30–65% of appendicitis cases involve retroperitoneal location [4, 5]. Studies have shown that retrocecal location, which is closely connected to the retroperitoneum, is the most common site of appendiceal perforation [6, 7]. Severe complications resulting from appendicitis are rare but can have serious consequences. Nahhas reported a case of retroperitoneal abscesses caused by retroperitoneal appendix perforation, and poor drainage in such case lead to bad recovery after surguey [8].

The retroperitoneum contains potential spaces such as the posterior pararenal space, anterior pararenal space, and perirenal space [9]. These spaces are filled with adipose tissue. When perforation occurs in the retroperitoneal region of the digestive tract, these spaces become more vulnerable than the peritoneum, making it challenging for surgeons to effectively clean and drain during and after surgery. In our case, we placed multiple drainage tubes to aid in eliminating sepsis, and the tubes were effective.

Retroperitoneal perforation is an uncommon event but carries a high mortality rate of ~20% [10]. Reported cases of retroperitoneal abscesses caused by colonic diverticular or cecal perforations have resulted in septic shock and death shortly after surgery [11]. The spread of air or pus in the soft tissues of the retroperitoneal space follows a specific pattern, and the body exhibits distinct signs [12]. In our case, pus spread from the space behind the cecum along the mesentery to the stomach, causing back pain and indicating the interconnectedness of these regions. Other reported cases have demonstrated connections between the retroperitoneum and various parts of the body, leading to complications such as retroperitoneal necrotizing fasciitis [13], thigh abscesses [14], and scrotal abscesses [15]. It has been observed that the visceral space extends from the neck through the mediastinum to the retroperitoneum, forming an anatomical connection between these areas [16]. Perforation into the retroperitoneal space can result in pneumomediastinum and cervical subcutaneous emphysema [17], which are more severe than the symptoms observed in our case.

## Conclusion

The management of typical appendicitis follows a standard approach. However, when the retroperitoneum is involved, a different strategy may be required. The retroperitoneum is a complex space with interconnected compartments. Cleaning up when it is filled with contaminated material can be challenging. Adequate postoperative drainage significantly reduces the risk of septic shock. When dealing with a retroperitoneal abscess caused by perforation, addressing the perforation is the first priority. While necrosectomy and debridement are crucial, ensuring proper drainage is of even greater significance.
